# Awake Prone-Positioning in Patients on Non-Invasive Ventilation for Management of SARS-CoV-2 Pneumonia: A Systematic Review

**DOI:** 10.3390/arm90040046

**Published:** 2022-08-18

**Authors:** Geetanjali Tolia Chilkoti, Medha Mohta, Zainab Ahmad, Ashok Kumar Saxena

**Affiliations:** 1 Department of Anesthesiology and Critical Care, Guru Teg Bahadur Hospital, University College of Medical Sciences, Shahdara, Delhi 110095, India; 2 Department of Anesthesiology and Critical Care, All India Institute of Medical Sciences Bhopal, Saket Nagar, Bhopal 462020, India

**Keywords:** awake prone positioning, non-invasive ventilation, helmet NIV, COVID-19

## Abstract

**Highlights:**

- Awake proning has an established role in both COVID and non-COVID acute hypoxemic respiratory failure (AHRF).
- Patients with COVID AHRF are often on NIV, and the use of awake prone positioning may pose concerns regarding feasibility, efficacy and side effects.
- This systematic review was undertaken to evaluate the feasibility and efficacy of awake proving along with NIV in NIV patients.
- Awake proning in NIV patients had a feasibility ranging from 36 to 100% and an increased efficacy in terms of improvement in oxygenation with no significant side effects.
- We observed a moderate-to-serious risk of bias amongst the included non-randomized observational studies and heterogeneity in terms of respiratory supports and/or the interface used by the patients.

**Abstract:**

Introduction: Patients with corona virus disease-19 (COVID-19)-induced acute hypoxemic respiratory failure (AHRF) are often on non-invasive ventilation (NIV) and use of awake prone positioning (PP) may pose concern in terms of feasibility, efficacy and side effects. This systematic review was undertaken to evaluate the feasibility and efficacy of awake PP along with NIV in them. Materials and Methods: A systematic literature search was conducted from the inception of COVID-19 until 15 August 2021. Various factors including feasibility, interface used, outcome, efficacy, side effects and limitations in both intensive care unit (ICU) and Non-ICU setups were noted. Results: A total of 12 original articles and six case series including 359 patients were involved. Out of it, 40% (n = 122) of patients were in ICU and 60% (n = 237) in Non-ICU areas. Four clinical studies and four case series including 114 patients had evaluated PP along with helmet continuous positive airway pressure (CPAP). All had found PP with helmet CPAP to be feasible and efficacious; however, only one study documented the sustained improvement in oxygenation i.e., 12 h after PP. Conclusions: The present systematic review observed moderate to serious risk of bias amongst the included studies along with heterogeneity in terms of varied respiratory support amongst patients. However, the use of awake PP in patients on NIV has been found to be feasible and efficacious with no adverse events.

## 1. Introduction

Prone positioning (PP) has been an established technique for improving oxygenation in patients with severe acute respiratory distress syndrome (ARDS) on mechanical ventilation [1,2]. Considering the proven benefits of PP in intubated patients, it was also assumed to have improved oxygenation in awake, non-intubated patients with acute hypoxemic respiratory failure. The outbreak of the corona virus disease-19 (COVID-19) pandemic led to the widespread use of awake self-prone positioning for treatment of moderate to severe acute respiratory syndrome coronavirus (SARS-CoV-2) infection [3]. It has also been accepted by the United Kingdom Intensive Care Society (UK-ICS) as a standard of care for suspected or confirmed COVID-19 patients requiring an FiO_2_ ≥ 28% [4]. Awake self proning is a low-risk intervention requiring minimal assistance and has been enormously applied in both intensive care unit (ICU) and Non-ICU setups worldwide [5].

Patients with moderate to severe COVID-19 induced acute hypoxemic respiratory failure (AHRF) often require non-invasive ventilation (NIV) for management. Non-invasive ventilation is less resource intensive than invasive ventilation and has the advantage of being more patient compliant and can be managed outside of critical care setup. Although NIV is utilized widely in the management, more research is needed to elucidate its benefits and risk associated with viral transmission via droplets [6]. However, it may help in obviating the need of invasive ventilation, a highly infectious procedure in terms of the risk of viral transmission to health care workers [7]. Considering the proven benefits of awake PP in COVID-19 disease, its use has also been tried in patients receiving NIV.

As COVID-19 is a novel viral disease and the evidence available so far to support the efficacy of awake PP in NIV is limited, an imperative concern with its use in COVID-19 induced AHRF is if awake PP is feasible, beneficial or if associated with side effects. The purpose of this systematic study was to evaluate the feasibility, efficacy and side effects of awake PP in patients receiving NIV admitted to both intensive care unit (ICU) and Non-ICU set ups.

## 2. Literature Search and Data Source

We conducted a comprehensive literature search using PubMed, MEDLINE, EMBASE and Google Scholar from December 2019 till 15 August 2021. In PubMed, the following search strategy was used: “(COVID-19 OR Novel Coronavirus–Infected Pneumonia OR 2019 novel coronavirus OR SARS-CoV-2) AND (prone oxygenation OR awake prone position OR self proning) AND (Non-invasive ventilation OR Continuous positive airway pressure OR Helmet continuous positive pressure). The strategy was then further adapted for the other databases. The titles and abstracts of each article were further reviewed to evaluate their relevance to our study. Full-text articles were retrieved for further consideration for inclusion. Two authors (G.T.C. and Z.A.) read all the articles and any inconsistencies were resolved by consensus with the third author (M.M.).

For study selection, we followed PICO framework: Participants; who had a validated diagnosis of COVID-19, irrespective of stage or severity of disease receiving NIV with either of the interface i.e., continuous positive airway pressure (CPAP) mask/Helmet NIV; Intervention: awake self proning in these patients; Comparison: patients on NIV but not receiving PP, if an original article; Outcomes: various parameters indicating feasibility, oxygenation or side effects. A priori, both interventional and observational data were considered. Considering the novelty of the topic, we have incorporated case reports and case series in addition to original articles providing evidence towards the efficacy of awake PP in improvement of oxygenation in COVID-19 along with NIV. We did not impose any language restriction in order to include maximum articles and minimize language bias. For each article, we extracted data regarding authors, year of publication, the period of observation, NIV interface used, ICU or non-ICU setting, duration of PP, outcomes assessed, efficacy, conclusions and limitations, if any.

## 3. Results

**Figure 1:**
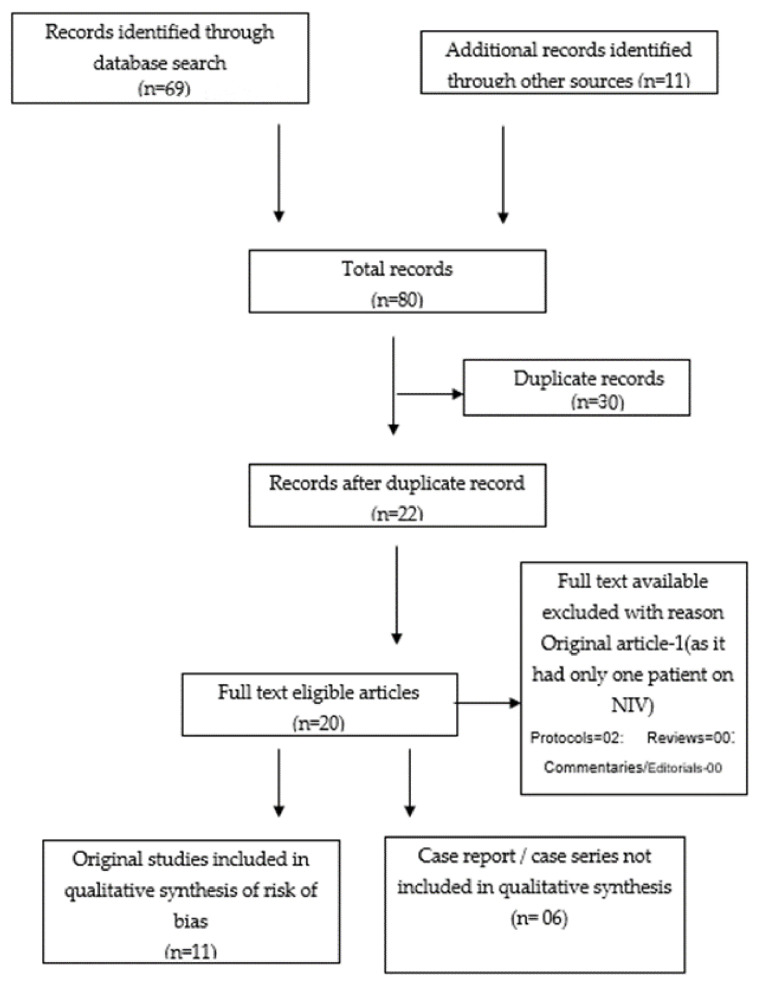
PRISMA flowchart depicting the steps of qualitative synthesis of evidence from the literature search.

For literature search and following the screening of titles, abstracts and removal of duplicates, we retrieved 12 original articles, [8,9,10,11,12,13,14,15,16,17,18,19] six case series [20,21,22,23,24,25,26] and two protocols [27,28] (Figure 1). We could not find any review article addressing awake PP in COVID-19 patients exclusively on NIV. Finally, 12 original articles [8,9,10,11,12,13,14,15,16,17,18,19] (Table 1) and six case-series [20,21,22,23,24,25,26] (Table 2) involving a total of 359 patients receiving awake PP along with NIV were included. Out of a total of 359 patients on NIV for which awake PP was evaluated, 40% (n = 122) of patients were in ICU and 60% (n = 237) in Non-ICU areas including emergency areas, respiratory high dependency unit (HDU), etc. Out of 12 original articles, one was excluded as it had only one patient on NIV [19]. Finally, 11 original articles with a total of 308 patients were included for qualitative analysis.

### 3.1. Awake PP along with NIV in Patients with COVID-19 Pneumonia in Different Setups i.e., ICU and Non-ICU Setups

The majority of the studies are conducted in the Non-ICU setups i.e., HDU, Respiratory ward or HDU and Emergency ward [8,9,10,11,12,14], whilst only a few studies were conducted in the ICU setup [13,18,19]. In addition, four clinical studies evaluating awake PP in patients on Helmet NIV were conducted in Non-ICU setups [9,11,16,18].

Amongst all studies in the ICU setup [13,18,19], a prospective multicentre, open-label, parallel arm, randomized clinical superiority trial evaluated awake PP in both NIV and high flow nasal cannula (HFNC) patients and observed that 33% of patients were intubated in both of the groups [18]. Ripollo-Gallardo et al. [20] and Bastoni et al. [21] in their retrospective series of 13 and 10 patients evaluated PP amongst patients on helmet NIV in general ward and emergency department, respectively. Both studies showed improved oxygenation with PP; however, Bastoni et al. [21] also conducted lung USG after one hour of PP but did not observe any change in recruitability (Table 2). As far as the feasibility of PP with helmet NIV is concerned, it was reported as 92.3% by Ripollo-Gallardo et al. [20] and 60% by Bastoni et al. [21]. In a similar study on patients with helmet CPAP by Golestani et al. [22], an improvement in P/F ratio was observed even after 12 h in 30% of patients. The tripod position with Helmet CPAP [23] and lateral positioning (LP) with CPAP mask [24] have also been found to be feasible and effective in improving oxygenation. One case report also used dexmedetomidine to assist with compliance with awake PP in patients on NIV [25]. Similarly, a case series of 13 patients with variable respiratory support also observed that awake PP is feasible and effective [26].

### 3.2. Awake PP with Helmet CPAP

Only four clinical studies [8,10,15,17] and four case series [20,21,22,23,24] including 114 patients have evaluated PP along with helmet CPAP. All the four case-series found PP with helmet CPAP to be feasible and efficacious [28,29,30]; however, the sustained improvement in oxygenation even after 12 h of PP was documented only by one study [10] Out of four clinical studies, two were conducted exclusively in patients receiving Helmet CPAP [10,15], whereas the remaining two included Helmet CPAP along with COT (conventional oxygen therapy) [8] and HFNC [17].

In the very first pilot observational prospective study on 26 patients receiving NIV helmet CPAP trials, a significant improvement of gas exchange was observed along with a clinically relevant decrease of A-a O_2_ by <20% with the use of PP vs. lateral positioning [10]. The success rate was higher with PP than LP. On the contrary, Paternoster et al. evaluated PP as a rescue strategy exclusively in patients after failing the CPAP trial in a supine position (with PF < 150) [15]. They described their experience of use of PP along with helmet CPAP and also reported the use of dexmedetomidine to improve patient compliance. Out of 11 patients, only two were non-compliant depicting a low failure rate of 27%; in addition, the 28-day survival rate was high, i.e., 82%. This case series concluded PP to be feasible and without complications in patients with AHRF when applied along with dexmedetomidine infusion [15].

Similarly, a prospective cohort study assessed the feasibility of PP in 56 patients receiving NIV or conventional oxygen therapy (COT) and found it to be feasible in 83.9% of patients (n = 47) Oxygenation improved from the PaO_2_/FiO_2_ ratio 180.5 mmHg to 285.5 mmHg (112.9) in PP (*p* < 0.0001). Oxygenation following resupination was maintained in only 23 patients (50%). Thirteen patients were intubated, and five deaths were observed at follow-up. It was concluded that PP was feasible and effective in improving oxygenation in awake patients with COVID-19; however, the effect was sustained in only 50% of patients. Simoli et al. observed that PP was tolerated better in patients with full CPAP masks then helmet CPAP masks. They also concluded that PP works best when applied early and for at least 10 h/day [17].

Interestingly, all aforementioned case studies and case series were conducted in Non-ICU set ups including the HDU and emergency ward, except for a single case series in the ICU setup [22].

Feasibility: As far as the feasibility is concerned, most of the studies found awake PP to be feasible along with NIV ranging between 36% to 100%. However, the duration of proning is an important factor determining the feasibility. Two studies reported a very high feasibility of 83.9–100% when PP was attempted for 3 h only [8,9], whereas, on increasing the duration of PP to 12 h, only 6% of patients were observed to be compliant [18]. Few studies reported it to be 100% irrespective of the duration [12,15,16]. One study evaluating a semiprone position in NIV observed that proning was achieved in 36.7%, semiproning in 56.7% of patients, and absolute noncompliance in 6% of patients. The major reasons cited for non-feasibility were discomfort, coughing, non-cooperation, and worsening oxygenation [11].

Efficacy: Various parameters have been used to assess the efficacy of awake PP in NIV patients. It includes SpO_2_, P/F ratio, ROX index (Ratio of SpO_2_/FiO_2_ to respiratory rate) [8,9,14,15,16,17], reduction in alveolar-arterial gradient (A-a O_2_) [10], reduction in respiratory rate (RR) and endotracheal intubation (ETI) rate [12,13,18,19], 28-day mortality [11]. Most of the studies have used the P/F ratio, with SpO2 as the oxygenation parameter to evaluate the efficacy, whereas few have used intubation rate and death rate as the definite outcomes. As far as the intubation rate is concerned, few studies reported a reduction in intubation rate with the use of PP [12,13,18], whereas few did not report any difference [19,20]. A multicentre observational trial did not observe any improvement in the risk of intubation or 28-day survival with the use of PP in 40 patients receiving oxygen therapy via various modes including COT, NIV and HFNC [19]. However, in this study, only one patient was receiving PP along with NIV. Rosen et al., in addition to no difference in the intubation rate, also reported higher mortality with PP than the control group i.e., 17% vs. 8% [18].

Complications: None of the studies reported any complication with awake PP along with NIV except for a multicentre RCT by Rosen et al. [17] They had reported pressure sores in 6% of patients in PP group when compared to 23% in the control group. Except this, none of the studies have reported any serious adverse event with the use of awake PP in patients receiving NIV.

### 3.3. Risk of Bias (Quality) Assessment

**Figure 2:**
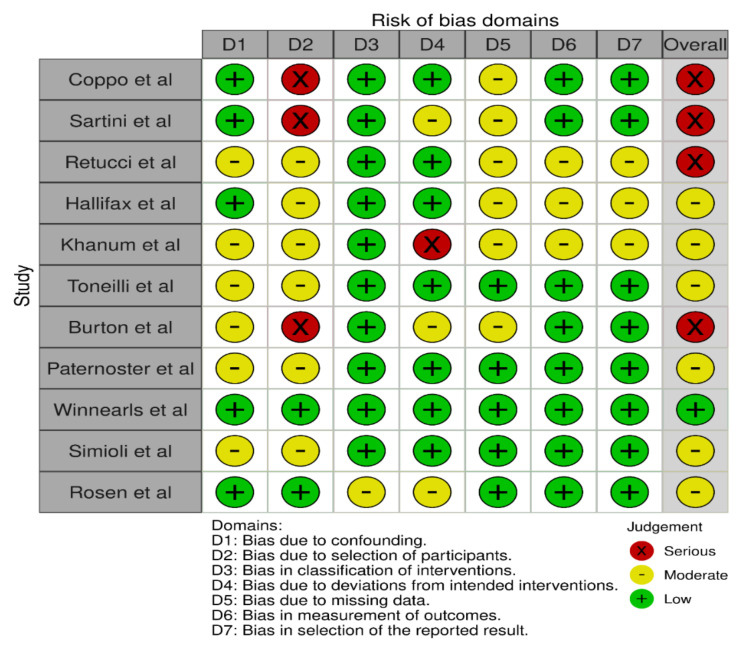
Traffic light plot for risk of bias assessment using the ROBINS-I tool [30].

**Figure 3:**
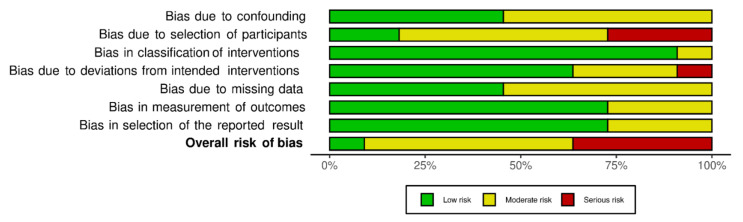
Weighted bar plot for risk of bias assessment using the ROBINS-I tool [30].

The Cochrane Collaboration tool, namely ROBINS-I (“Risk of Bias In Non-randomised Studies—of Interventions”), was used to assess the risk of bias of the included studies. It is a tool for evaluating risk of bias from non-randomised studies utilizing interventions. This tool assesses risk of bias in seven domains i.e., bias due to confounding, bias due to selection of participants, bias in classification of interventions, bias due to deviation from intended intervention, bias due to missing data, bias in measurement of outcomes, and bias in selection of the reported results. Each aforementioned parameter of bias in each study will be scored as having low, medium, high, or unclear risk. The study with lower risk is deemed as a high-quality study. Risk of bias was independently assessed by GCT and ZA, and disagreements were resolved through discussion with MM. The overall judgement on the bias assessment following assessment of each domain of the included studies in the present systematic review as per ROBIN-I tool has been found to have moderate to serious risk. A retrospective observational multicentric trial by Jouffroy et al. was not included for qualitative synthesis of risk of bias as, out of 40 patients who received PP, only one patient was on NIV and the rest were all either on HFNC or on COT [19]. The risk of bias was variable among different included studies. The traffic light plot and weighted plot depicting different biases of all the included non-randomized studies using the robvis web app are shown in Figure 2 and Figure 3, respectively [29]. The overall judgement on the bias assessment following assessment of each domains of the included studies in the present systematic review as per ROBIN-I tool has been found to have moderate to serious risk [30].

## 4. Discussion

The present systematic review has summarized the current evidence of awake PP in patients with SARS-CoV-2 on NIV. Out of a total of 359 patients on NIV in whom awake PP was evaluated, 40% (n = 122) of patients were in ICU and 60% (n = 237) in Non-ICU areas including emergency areas, respiratory HDU, etc. Overall, the technique was found to be feasible (36–100%) and efficacious in almost all of them with minimal to no side effects. However, the assessment of risk of bias of the included studies is observed to have moderate to serious risk.

Prone positioning has been recommended for intubated and mechanically ventilated patients. Literature is replete with the articles addressing the advantages of awake self PP in COVID-19 [31,32]. Considering its benefit in awake patients with COVID-19, it seems reasonable that comparable benefits in terms of improved oxygenation, lesser invasive ventilation and improved overall outcomes may be achieved in patients on NIV.

The COVID-19 pandemic has witnessed an increasing use of NIV for management of AHRF as it decreases the need for intubation leading to decreased morbidity and mortality and thus reducing the need for ventilator availability, especially in the geographical areas hit hard by the pandemic. The use of NIV and HFNC in immunocompromised patients suffering from AHRF have caused lower intubation rates, lower mortality and a reduced length of ICU stay [33,34]. The HFNC is another non-invasive strategy that gained popularity during these COVID times. However, its role has been controversial, and there is no consensus in this context for the use of HFNC in COVID-19 pneumonia amongst various eminent organizations [35]. Considering this, we restricted this systematic review to NIV only.

In moderate to severe COVID-19 disease, there is inhomogeneity in the lungs, and CT scans of COVID-19 patients typically show a ground glass appearance [36]. Prone positioning helps to drain secretions from the lung peripheries and improve lung dyshomogeneity and recruitment, thus leading to improved ventilation/perfusion match.

A pertinent concern is whether PP can be considered as a ‘preventative’ adjunct rather than ‘rescue’ therapy in ARDS. The preventive use of PP can delay or avoid mechanical ventilation, thus reducing associated morbidity and mortality. All studies had used awake PP as an adjunct along with NIV to improve oxygenation. However, only Paternoster et al. [15] had used PP as a rescue treatment in patients with acute hypoxemic RF if saturation did not improve after one hour of a CPAP helmet trial. For rescue treatment, PP was given for 12 h followed by 6 h in supine position along with helmet CPAP. The study clearly showed an improved 28-day survival rate i.e., 82% and low failure rate i.e., 27% with the use of awake PP as a recue modality in patients with COVID-19 induced AHRF.

As far as the interface for NIV is concerned, it has been observed that the helmet CPAP mask is superior to the face mask in terms of tolerability and PEEP titration and there is a reduction in the need for endotracheal intubation [37] without affecting functional independence at hospital discharge along with less mortality and more functional independence at one year compared to NIV [38]. Similarly, in the current scenario of administering NIV along with awake PP in COVID-19 patients, we observed helmet CPAP to be feasible and efficacious.

Feasibility is an important concern with the use of PP along with NIV, more so with the use of helmet CPAP mask. Out of the aforementioned studies evaluating PP in patients on the helmet CPAP mask, only Paternoster et al. had used dexmedetomidine for IV sedation. This study had reported 100% feasibility and a high survival rate. Dexmedetomidine, a centrally acting alpha 2 agonist, has sedative and anxiolytic actions without any respiratory depressant actions; however, in this situation, it may lead to improved patient compliance. Recently, Salcedo et al. have effectively used dexmedetomidine to assist with compliance and tolerance during alternating supine prone cycles during self proning in a COVID patient on alternating NIV and HFNC [25].

However, Chad et al. [5] and Chilkoti et al. [31] in their reviews implicated this short-term improvement in oxygenation with awake PP to be simply a ‘recruitment manoeuvre’ and claimed it to be efficacious in only patients with mild to moderate disease. In the present systematic review, the short-term improvement in the oxygenation amongst included trials could simply be a “recruitment manoeuvre” as a majority of the oxygenation outcome parameters were assessed between a few minutes to hours after PP in various studies. However, only one study by Simioli et al. [17] has evaluated oxygenation parameters after 10 h of initiating PP along with NIV. As far as the duration of PP is concerned, Simoli et al. also recommended the early use and a minimum of 10 h/day of PP in patients with NIV. They had also emphasized the interface fit for better outcomes [17].

The present systematic literature review deals with a few limitations. Firstly, the studies included had small sample sizes and single centric data, and the majority were retrospective in nature. Secondly, except for two [18,19], all studies lacked the control group. Interestingly, all case studies and case series of NIV along with helmet CPAP were conducted in Non-ICU set ups including HDU and emergency ward, except for one case series by Golestani et al., which was conducted in ICU setup, a finding which cannot be related in developing countries like ours with limited facilities during these pandemic times. Thirdly, few studies incorporated heterogeneous patient population with variation in respiratory support/interface used at the time of initiation of PP.

## 5. Conclusions

In conclusion, the literature available so far encourages the use of early awake self proning for management of SARS-CoV-2 infection in patients on NIV. We observed feasibility ranging between 36–100% and increased efficacy in terms of improvement in oxygenation with no significant side effects. The gathered evidence pertaining to the use of awake PP along with NIV for management of COVID-19 disease is not sufficient. We observed moderate to serious risk of bias amongst the included non-randomized observational studies and heterogeneity in terms of respiratory supports and/or interface used by the patients. Further well-designed multi-centric studies with a larger sample size and preferably with a control group are warranted to evaluate the awake PP as an adjunct in patients on NIV for management of COVID-19 pneumonia in terms of its feasibility, optimal duration of proning and long-term efficacy in improving oxygenation.

## Figures and Tables

**Table 1:** Characteristics of all the clinical studies evaluating awake PP in patients with COVID-19 pneumonia on NIV.

Authors	Type of Study/Single or Multicentric	Set Up	N	Mean Age (SD/IQR) of Patients in PP	Mode of Oxygen Therapy Interface Used	Measure/ Outcomes and Their Interval of Measurements	Duration of Proning	Feasibility	Efficacy	Complications	Conclusion	Limitations
Coppo et al., 2020 [8]	Single centre, prospective cohort, feasibility study, Single centric	Ward/ED/Resp HDU	56	57·4 (7·4)	Patients on either Helmet CPAP or COT	PaO_2_/FiO_2_ at 10 min after PP and 1 h after resupination; Safety, Feasibility, PaCO_2_	Initially 3 h, up to 7 h	Feasible in 83.9% of patients (i.e., for at least 3 h). Unfeasible in rest due to discomfort, coughing, non-cooperation, worsening oxygenation	ETI rate similar in responders vs. non-responders (26% vs. 30%) P/F ratio increased after PP (180.5 mm Hg vs. 285.5 mm Hg). 50% were labelled ‘’ responders’’ who maintained oxygenation after resupination	-	Prone positioning in awake, spontaneously breathing patients is feasible outside of the critical care environment in most patients.	Lack of control group; lack of randomisation, Selection bias; Single centre data
Sartini et al., 2020 [9]	Cross-sectional, before-after study Single centric	Non-ICU	15	59 (SD 6.5)	Patients on NIV	SpO_2_, P/F ratio, RR, and patients’ comfort were assessed at 3 time points while on NIV in PP i.e., baseline, at 60 min after starting NIV, and after 60 min of end of NIV session	Median number of NIV cycles in the PP was 2 (IQR- 1-3 cycles) for a total duration of 3 h (IQR: 1-6 h)	Feasible in all	Significant reduction in RR both during and after pronation; significant improvement in SpO_2_ and P/F during pronation in all. Sustained improvement in SpO_2_ and PaO_2_/FiO_2_ in 80% (n = 12); unchanged 13.3% (n = 2); and worsened in 6.7% (n = 1)	-	NIV in the prone position to patients with COVID-19 and ARDS on the general wards was feasible with higher oxygenation was higher during and after pronation.	Small sample size; Short duration of NIV in PP; No control group; Selection bias
Retucci et al., 2020 [10]	Pilot, observational, & prospective study Single centric	Resp HDU	26	62 (IQR: 56-69);	Patients on helmet CPAP treatment with P/F ratio <250 for more than 48 h	(Assessment in both PP and Lateral position) Success of proning trial i.e., a decrease of the A-a O_2_ gradient of at least 20%; Equal or reduced RR and dyspnoea; SBP ≥90 mm Hg	One hour	Feasible in 92% (n = 24), not feasible in two, reason was discomfort	Among trials in PP, 33.3% succeeded; 41.7% showed decreased A-a O_2_ by <20%, and 25% failed. Among trials conducted in lateral positioning, 8% succeeded; 52% showed decreased A-a O_2_ by <20%, and 40% failed	-	Prone positioning had greater benefit than lateral position in patients on NIV The increase in A-a O_2_ was <20% and was not sustained in the semi-recumbent position.	Did not assess the clinical outcome or confounders such as FiO2 or length of CPAP trial before PP; Evaluation of response was conducted after only one hour
Hallifax et al., 2020 [11]	Retrospective study Single centric	Resp HDU	Total 48 PP-30	69 (IQR 54-80)	Patients on CPAP/HFNC	Feasibility Death with PP	>2 h, twice daily for at least 2 days	Proning was achieved in 36.7% of patients and semiproning in 56.7% of patients. 6.7% of patients refused proning after initial attempt	Achievement of full PP associated with lower mortality than failed or semi-proning (0.0% vs. 63.2%.)	-	Patients on CPAP more likely to be able to successfully prone than those on HFNC (52.9% vs. 15.4%)	Potential selection bias, lack of control group
Khanum et al., 2021 [12]	Retrospective, observational study Single centric	Special Care Unit	23	54.5 (SD 11.7)	Oxygen therapy with or without NIV	Avoidance of intubation, mortality, length of hospital stay	No prefixed targeted duration but for >1 day	Feasible in all patients	Only one patient required intubation and died, rest 22 improved after 3–5 days Mean hospital stay 12 days.	-	All discharged home on room air or minimal oxygen requirement	Small and heterogenous sample size, no defined duration of PP, no control group
Tonelli et al., 2021 [13]	Retrospective observational study Multicentric cohort	ICU	114 (Standard treatment = 76 PP = 38)	PP = 61 (IQR 32–75) Standard treatment = 70 (IQR 33-80)	Patients either on NIV/HFNC/CPAP	Intubation rate, in-hospital mortality, time to intubation, tracheostomy, length of ICU and hospital stay	3 h (1–4 sessions/day)	-	Reduced intubation rate with PP (18% vs. 39.5%, Less time spent in PP independently associated with IMV. Respiratory support free days with PP vs. standard (20 vs. 15), length of ICU (10 vs. 15 days) and hospital stay (20 vs. 24 days) shorter in PP than standard care	-	PP significantly reduces intubation rate in patients on HFNC but not NIV or CPAP. Time to intubation, tracheostomy, mortality rates did not differ between standard care and PP groups	Different SOPs in both centres; duration of PP was variable; non-randomised sample; patients in PP group was significantly younger
Burton et al.2020 [14]	Retrospective observational study Single centric	ICU	20	53.4 ± 8.3	Patients on NIV (CPAP mask)	P/F ratio, changes in HR & RR before, during and after proning	5 cycles (IQR 6.3) with mean duration of 3 h (IQR 2)	Feasibility in 65% (7 out of 20 non-compliant)	P/F ratio increased by 28.7 mm Hg, no significant change in HR, RR was noted	-	PP in conjunction with NIV can improve oxygenation without significant adverse effect	Median age <60 yr (results can not be translated to older age group), small sample size, no set criteria for PP or intubation
Paternoster et al. 2020 [15]	Retrospective observational single centric study	HDU	11	62 (10)	Helmet CPAP in prone after failing CPAP trial in supine position (PF < 150)	P/F ratio, SpO2, RR baseline, then after 24, 48 and 72 h of PP	12 h proning followed by 6 h supination	Feasible in all patients. Sedation (Dexmedetomidine) improved comfort. Mean duration 7 ± 2.7 days	P/F increased from prior to proning 10.75 ± 20.8 to 244.4 ± 106.2 after 72 h of proning (*p* < 0.001), SpO2 increased from 90.6 + 2.3 to 96 + 3.1 (*p* < 0.001). RR decreased from 27.6 ± 4.3 to 20.1 ± 4.7 after 72 h.	-	27% of patients required IMV Overall 28-day survival rate was 82%.	Non -randomized, small sample size, no control group
Winearls et al. 2020 [16]	Retrospective study Single centric	Resp HDU	24	62 (13)	CPAP full face mask	P/F ratio & ROX index at baseline, 15 min after PP initiation, one hour after PP while on CPAP	8 ± 5 h in first 24 h (mean of 10 ± 5 days)	Feasibility- 92% (2 failed to tolerate PP; 12 able to fully prone, 10 semiprone)	ROX index (7.0 ± 2.5 baseline vs. 11.4 ± 3.7 on CPAP with PP) and P/F ratio increased significantly on CPAP with PP (from 252 ± 87 mm Hg vs. 252 ± 87 mm Hg). Increase maintained only for one hour after cessation of PP	No complications	PP along with CPAP therapy is feasible, safe and improves oxygenation No difference in outcome with fully prone vs. semiprone position	Non-randomized, no control, small sample size, no defined proning protocol
Simioli et al., 2021 [17]	Retrospective Case control study Single centric	Resp HDU	29	64 (±22.5)	Helmet/CPAP full face mask/HFNC (10 Helmet CPAP, 13 full CPAP face mask, and 6 HFNC)	P/F ratio at baseline, 2 h after NIV and after 10 h of initiating PP feasibility	At least 10 h/day with cycling every 2 h alternately between prone, right and left lateral and semiupright position	Feasibility—62% (11 patients were noncompliant Tolerability better in CPAP full face masks than helmet. Causes of non-compliance are interface displacement, oxygen desaturation, worsening of dyspnea, chest tightness, neck pain, and agitation	P/F ratio increased significantly during PP (288 ± 80 vs. 202 + 122), duration of respiratory failure significantly shorter with PP (median 14 vs. 21 days). Need for IMV less in PP group (5.5% vs. 18%)	No complications	PP along with NIV efficacious when started early and for at least 10 h/day.	Small sample size
Rosen et al., 2021 [18]	Randomised clinical trial Multicentric trial	-	75 (36-PP 39-Controls)	65 (53–74)	HFNC/NIV with P/F ratio ≤ 20 kPa or corresponding values of SpO2 and FiO2	Intubation within 30 days after enrolment	At least 16 h PP per day. Prone and semi-PP allowed	Feasibility-17% (Only 6% of patients able to adhere to the 16 h of proning as defined in this study	30-day intubation rate not different between cases with PP and controls. (33 & vs. 33%)	6% of patients had pressure sores in PP group vs. 23% in control group.	No difference in intubation rate, ventilator free days, days free of NIV/HFNC, hospital or ICU stay or organ support with the use of PP. 8% of deaths in control group vs. 17% in PP group.	non blinded
Jouffroy et al., 2021 [19]	Retrospective observation study Multicentric trial	ICU	Total 379 patients, 40 PP	59.5 (56; 64)	HFNC/CPAP/COT (37/1/2)	Intubation within 28 days; mortality within 28 days; rate of ICU discharge	PP for 3–6 h, twice a day	Feasible	After adjusting for factors, neither risk of intubation or 28-day survival showed benefit in PP	-	58% in the PP group were discharged alive without intubation, 40% required invasive ventilation	Limited proning duration

N—number of patients; PP—Prone positioning; ED—Emergency department; HDU—High dependency unit; NRBM—Non-rebreathing mask; HFNC—High flow nasal cannula; A-a o_2_ gradient—Alveolar–arterial gradient; RR—respiratory rate; SBP—systolic blood pressure; CPAP—continuous positive airway pressure; COT—conventional oxygen therapy; NIV—non-invasive ventilation; RR—respiratory rate; VV-ECMO-Veno—venous extracorporeal membrane oxygenation; ROX index—Ratio of SpO2/FiO2 to respiratory rate; R/I ratio—Recruitment-to-inflation ratio; IMV—Invasive mechanical ventilation; ETI—endotracheal intubation.

**Table 2:** Characteristics of the six case series evaluating awake PP in patients with COVID-19 on NIV [20,21,22,23,24,25,26]. The mean age of all 50 patients included in the case series was 64.0 yrs. The most commonly used clinical outcomes are P/F ratio (PaO_2_/FiO_2)_ ratio, SpO_2_, clinical improvement and oxygen requirements.

Authors	N/Gender	Age (Yrs)	Set-Up	Mode of Oxygen Therapy/Interface Used	Duration of Proning	Outcome	Conclusion
Ripoll-Gallardo et al., 2020 [20]	13 (85%, males)	66	General ward	Patients on helmet NIV CPAP	Maintained as long as patient tolerated	PaO_2_/FiO_2_; respiratory rate	Feasible in all; Improved P/F compared to baseline in 12 patients (*p* = 0.003); No difference was found in the RR before and after PP (*p* = 0.20)
Bastoni et al., 2020 [21]	10 (80% males)	73 (62–87)	Emergency department	Patients on helmet NIV CPAP with no clinical improvement	1 h	P/F ratio; Lung USG	Feasibility—60%; An improvement in P/F ratio from 68 ± 5 mm Hg to 97 ± 8 mm Hg after 1 h of PP in all; No change in B-line quantity and distribution in lung USG after 1 h
Golestani Eraghi M et al., 2020 [22]	10	-	ICU	Patients with P/F ratio <150 and on helmet NIV	9 h	PaO_2_/FiO_2_ ratio after 1 and 12 h of PP	Feasible in all: 60% of patients had sustained improvement in P/F ratio after 1 h; 30% of patients had delayed positive result and one patient was intubated
Rauseo et al. 2021 [23]	1 (Male)	77	ICU	Mild SARS-CoV-2 pneumonia on helmet CPAP	3 h cycles of semi recumbent and tripod position for three days	Improved P/F ratio, RR and hemodynamic	Tripod position can be safety applied with NIV
Sellmann et al. 2021 [24]	2 (males)	63 and 77	ICU	Severe refractory hypoxemia receiving NIV	-	Improved P/F ratio	Prone and lateral positioning is efficacious in improving oxygenation
Salcedo et al.2020 [25]	Male	73	ICU	Refractory hypoxemia on alternating HFNC and IPPV	4 h alternate cycles of supine and prone	Improved P/F ratio	Dexmedetomidine may be used effectively to assist with compliance and tolerance with the use of awake PP in patients on NIV
Kandasamy et al.2020 [26]	13 (4 male, 9 females)	40.7	ICU	Mild to moderate disease on NIV, HFNC, venturi mask, Nasal cannula	-	P/F ratio improved with PP from 328 ± 65 vs. 154 ± 52 mmHg; A-a O_2_ gradient improved from a median of 170.5 mm Hg (127.8309) to 49.1 mm Hg IQR (45.0, 56.6)	Favourable and feasible results

N—number of patients; PP—Prone positioning; NRBM—Non-rebreathing mask; HFNC—High flow nasal cannula; CPAP—continuous positive airway pressure; COT—conventional oxygen therapy; NIV—non-invasive ventilation; RR—respiratory rate; USG—Ultrasonography; (A-a) O_2_—Alveolar to arterial gradient.

## Data Availability

The data presented in this study are available on request from the corresponding author.
